# Selective COX-2 inhibitor continues to be a safe alternative in patients with nonselective NSAIDs hypersensitivity

**DOI:** 10.5339/qmj.2022.fqac.4

**Published:** 2022-04-04

**Authors:** Sherin Thalappil, Maryam Al-Nesf

**Affiliations:** ^1^Adult Allergy and Immunology Section, Department of Medicine, Hamad Medical Corporation, Doha, Qatar E-mail: SThalappil@hamad.qa

**Keywords:** celecoxib, COX-2 inhibitor, NSAID hypersensitivity

## Abstract

Background: Nonsteroidal anti-inflammatory drugs (NSAIDs) cause different types of allergic and pseudo allergic reactions. This results in difficulties in clinical practice. Most cases of NSAID hypersensitivity are mediated by the inhibition of cyclooxygenase-1 enzyme (COX-1), which results in depletion of the protective prostaglandin E2, and promotes the unrestrained synthesis of inflammatory mediators from mast cells. Selective COX-2 inhibitors are considered safe alternatives in patients with NSAID allergy, although hypersensitivity reactions to COX-2 inhibitors have also been reported. Our study aimed to report the experience in Qatar for using COX2 inhibitors as an alternative treatment for nonselective NSAID allergy.

Methods: Data of patients who underwent open challenge with a single dose of oral celecoxib 200 mg were retrieved from the procedure log of the Allergy and immunology Division in Hamad medical corporation, Doha, Qatar, from 2013 to 2022. The challenge was considered positive if the patient developed cutaneous or respiratory symptoms.

Results: A total of 31 patients were identified; 4 with a history of celecoxib allergy. The remaining 27 (23 females and 4 males); with mean ( ± SD, range) age of 42 ( ± 12, 20–65) years had hypersensitivity to one (n = 11) or more than one (n = 16) nonselective NSAID, manifested as cutaneous, respiratory, or anaphylactic symptoms. Those 4 patients with celecoxib allergy were challenged and only one with a historical reaction of anaphylaxis developed anaphylaxis during the challenge. Celecoxib was well tolerated in all 27 patients with hypersensitivity reactions to nonselective NSAIDs. Also, patients were contacted by telephone call at 24 hours and after 1 week with no evidence of delayed reactions.

Conclusions: Selective COX-2 and nonselective NSAIDs have similar overall efficacy as analgesic, anti-inflammatory, and antipyretic agents. Hypersensitivity reaction to COX-2 inhibitors has been reported; however, it is rare. So, it is safer to challenge the patients with COX-2 inhibitors before prescribing them as alternative medication in patients with Nonselective NSAID allergies. We plan to conduct a single-/double-blind placebo-controlled study for more patients, especially using graded challenges for high-risk profile candidates. Also, it may be of significance to test more than one type of selective COX-2 to avoid drug-specific reactions.

## Figures and Tables

**Figure 1.**
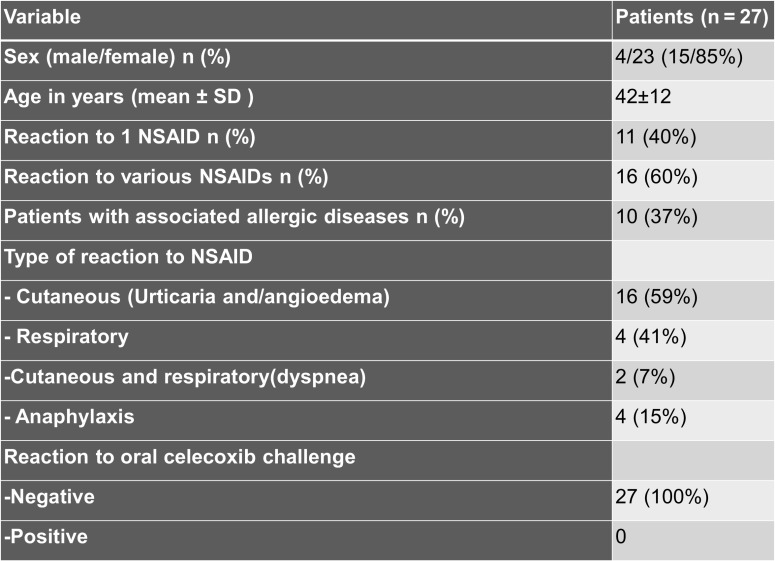
Delirium treatment algorithm.^3^ “a” off label use.

